# Supplementary material for: Causal Relationship between Aging and Anorexia Nervosa: A White-Matter-Microstructure-Mediated Mendelian Randomization Analysis
Source: Biomedicines. 2024 Aug 16;12(8):1874. doi: 10.3390/biomedicines12081874 (PMC11351342; doi:10.3390/biomedicines12081874)
Supplement: Supplementary file 1 [file biomedicines-12-01874-s001.zip › Supplementary Figure/Figure S4/Singe SNP plot of MR analysis for TL to ubm-b-1960.pdf]

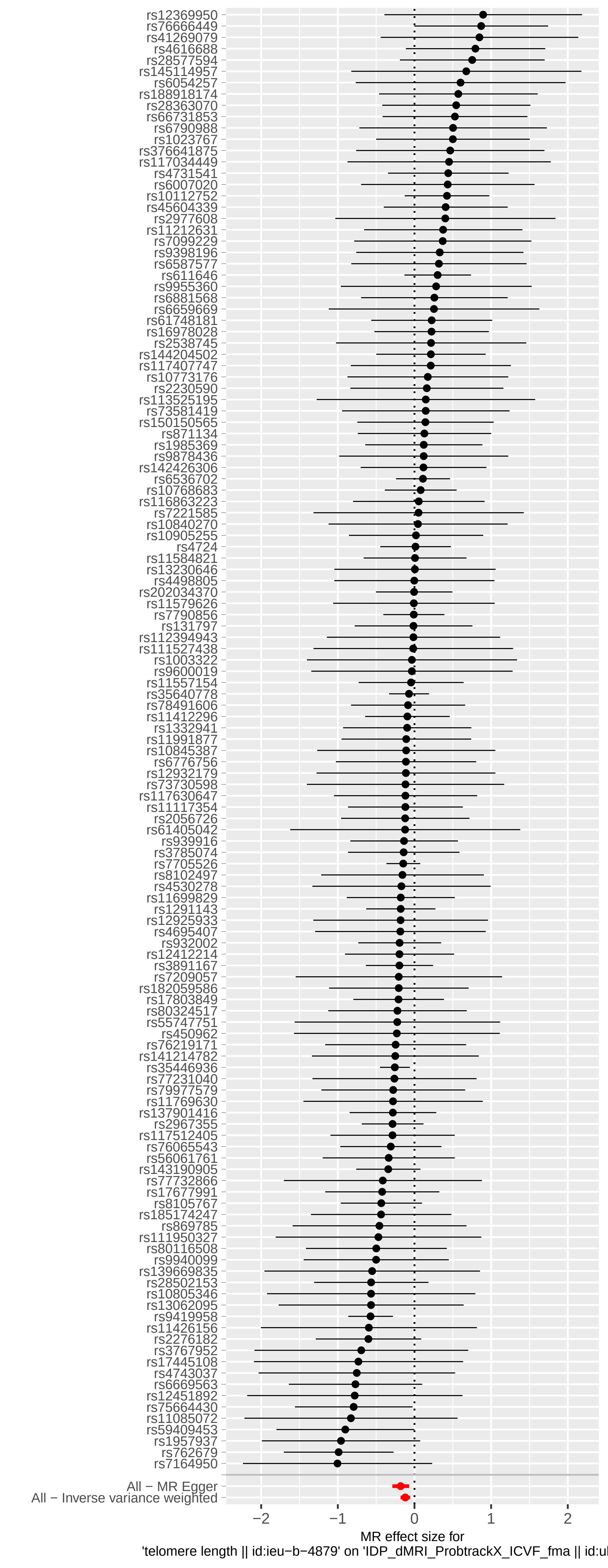
rs12369950
rs76666449
rs41269079
rs4616688
rs28577594
rs145114957
rs6054257
rs188918174
rs28363070
rs66731853
rs6790988
rs1023767
rs376641875
rs117034449
rs4731541
rs6007020
rs10112752
rs45604339
rs2977608
rs11212631
rs7099229
rs9398196
rs6587577
rs611646
rs9955360
rs6881568
rs6659669
rs61748181
rs16978028
rs2538745
rs144204502
rs117407747
rs10773176
rs2230590
rs113525195
rs73581419
rs150150565
rs871134
rs1985369
rs9878436
rs142426306
rs6536702
rs10768683
rs116863223
rs7221585
rs10840270
rs10905255
rs4724
rs11584821
rs13230646
rs4498805
rs202034370
rs11579626
rs7790856
rs131797
rs112394943
rs111527438
rs1003322
rs9600019
rs11557154
rs35640778
rs78491606
rs11412296
rs1332941
rs11991877
rs10845387
rs6776756
rs12932179
rs73730598
rs117630647
rs11117354
rs2056726
rs61405042
rs939916
rs3785074
rs7705526
rs8102497
rs4530278
rs11699829
rs1291143
rs12925933
rs4695407
rs932002
rs12412214
rs3891167
rs7209057
rs182059586
rs17803849
rs80324517
rs55747751
rs450962
rs76219171
rs141214782
rs35446936
rs77231040
rs79977579
rs11769630
rs137901416
rs2967355
rs117512405
rs76065543
rs56061761
rs143190905
rs77732866
rs17677991
rs8105767
rs185174247
rs869785
rs111950327
rs80116508
rs9940099
rs139669835
rs28502153
rs10805346
rs13062095
rs9419958
rs11426156
rs2276182
rs3767952
rs17445108
rs4743037
rs6669563
rs12451892
rs75664430
rs11085072
rs59409453
rs1957937
rs762679
rs7164950
All − MR Egger
All − Inverse variance weighted
−2
−1
0
1
2
MR effect size for
'telomere length || id:ieu−b−4879' on 'IDP_dMRI_ProbtrackX_ICVF_fma || id:ul
